# A critical view on the real-world security of logic locking

**DOI:** 10.1007/s13389-022-00294-x

**Published:** 2022-08-20

**Authors:** Susanne Engels, Max Hoffmann, Christof Paar

**Affiliations:** 1 Horst Görtz Institute for IT-Security, Ruhr University Bochum, Bochum, Germany; 2 Max Planck Institute for Security and Privacy, Bochum, Germany

## Abstract

With continuously shrinking feature sizes of integrated circuits, the vast majority of semiconductor companies have become *fabless*, outsourcing to foundries across the globe. This exposes the design industry to a number of threats, including piracy via IP-theft or unauthorized overproduction and subsequent reselling on the black market. One alleged solution for this problem is *logic locking*, where the genuine functionality of a chip is “locked” using a key only known to the designer. Solely with a correct key, the design works as intended. Since unlocking is handled by the designer only after production, an adversary in the supply chain should not be able to unlock overproduced chips. In this work, we focus on logic locking against the threat of overproduction. First, we survey existing locking schemes and characterize them by their handling of keys, before extracting similarities and differences in the employed attacker models. We then compare said models to the real-world capabilities of the primary adversary in overproduction—a malicious foundry. This comparison allows us to identify pitfalls in existing models and derive a more realistic attacker model. Then, we discuss how existing schemes hold up against the new attacker model. Our discussion highlights that several attacks beyond the usually employed SAT-based approaches are viable. Crucially, these attacks stem from the underlying structure of current logic locking approaches, which has never changed since its introduction in 2008. We conclude that logic locking, while being a promising approach, needs a fundamental rethinking to achieve real-world protection against overproduction.

## Introduction

In today’s semiconductor industry, many steps of the fabrication chain are outsourced for complexity and cost reasons. Most semiconductor companies have become *fabless*, with chip manufacturing, testing, and assembly performed at specialized providers across the globe. While avoiding the substantial costs of maintaining and upgrading own foundries, new threats arise when designs are sent to offshore fabs: integrated circuits (ICs) become susceptible to overproduction, counterfeit, and reverse engineering. Apart from the financial loss for semiconductor companies [1], counterfeited products can lead to major safety and security concerns [2].

In order to secure a design against rogue players in the fabrication chain, countermeasures such as *logic locking* have been proposed. Ever since, logic locking received a lot of attention by the scientific community, including publications in top security conferences, e.g., USENIX [3] and CCS [4]. The idea of logic locking is to integrate a locking mechanism into the circuit such that it produces faulty outputs whenever an incorrect key is provided. Only the holder of the intellectual property (IP) rights who is in possession of that key should be able to unlock the IC. Hence, although possessing all information required to fabricate the integrated circuit, a malicious entity lacks the secret key to unauthorizedly unlock ICs. Likewise, plain reverse engineering yields a locked design, i.e., depending on the scheme, the original IP can also be obfuscated to some extent. Hence, logic locking is regarded as a universal protection against *piracy*, of both physical nature (overproduction/counterfeiting) and logical nature (IP-theft).

In recent years, logic locking research has mostly become an arms race between increasingly specialized SAT-based attacks and corresponding countermeasures. This one-sided focus resulted in designs with strong SAT-resilience but serious design flaws that enable other far simpler attacks [5]. While the prospect of logic locking sounds promising, the lack of a common well-defined attacker model yielded many loosely argued security sketches that claim provable security but were broken shortly after.

The strong focus on SAT-based attacks and the inconsistencies in attacker models indicate that research on logic locking might need a restructuring. In this work, we systematically analyze these inconsistencies with a focus on the threat of overproduction, where a malicious entity in the fabrication chain obtains overproduced ICs and unlocks them for illegal reselling. Surveying existing work and the capabilities of modern foundries, we uncover practical attacks that invalidate the protection against overproduction of virtually all existing schemes, but lie outside of the current attacker models. Our argumentation provides strong indications that logic locking indeed needs a fundamental rethinking.

**Fig. 1:**
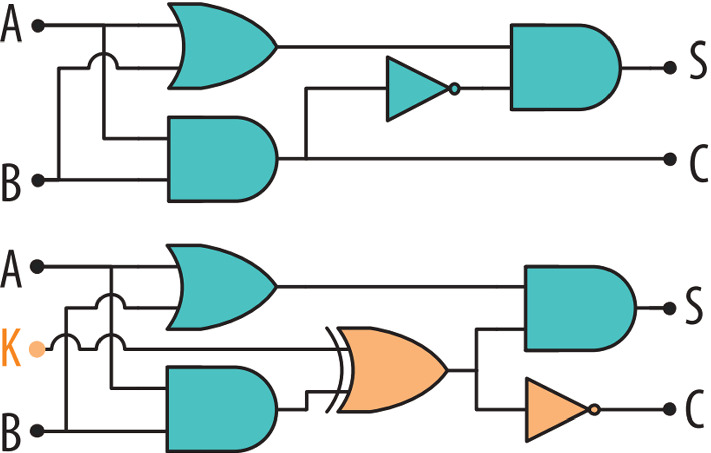
An example of logic locking with EPIC as depicted in [6]. On top is the original design and below the locked result

*Contributions* The work at hand provides four main contributions:

- We provide an up-to-date comprehensive survey of logic locking schemes, characterized by their locking approaches. Our survey is complemented with a systematic analysis of similarities and shortcomings of the attacker models and protection goals of previous work.
- We introduce a realistic attacker model for logic locking schemes that aim to protect against overproduction, which is not restricted to noninvasive attacks as in previous work. Our model is based on the real-world capabilities of a malicious foundry and, for a sound assessment of security, includes a precise definition of goals for a successful attack.
- We discuss novel attack vectors enabled by taking the identified capabilities of a malicious foundry into account. This uncovers two generic attack methodologies that target the foundations of all proposed locking schemes, based on *probing* and *minimal mask modification* as attack vectors. Notably and in contrast to existing works, we take (optional) initial key derivation, i.e., “key preprocessors,” into account.
- We extend our argumentation to generalize for virtually any logic locking scheme. Our discussion shows that potential countermeasures are merely hotfixes, the underlying vulnerabilities remain. The generic applicability of the attacks suggests that current logic locking will most likely never succeed against a determined adversary. As a conclusion, a major rethinking for logic locking approaches is imperative.

## Background on logic locking

In this section, we provide background information on logic locking. We introduce its motivation and main goals, before outlining how logic locking works in general.

### Protection goals

Most design houses have become *fabless*. Since outsourced processes are not within the direct control of the design house, they must be considered potentially malicious environments. Every external entity in the fabrication chain is hence *untrusted*.

Logic locking aims to protect an IC against piracy by untrusted parties in the fabrication chain, starting from the point when it leaves the design house, throughout the manufacturing process, and its remaining life cycle. Piracy can be of physical nature as in overproduced products, or IP-piracy through reverse engineering. The primary focus of logic locking is to prevent overproduction, i.e., only the design house should be able to control the (amount of) ICs available on the market. Some schemes, e.g., [4, 6–8], also claim general protection against IP-theft through reverse engineering or protection against hardware Trojan insertion. Note that these are *not* declared goals of all logic locking schemes and protection against these additional threats is not always provided, e.g., [9] only protects against overproduction.

### Locking procedure

Simplified, logic locking extends the existing design with a dedicated locking circuitry. This additional logic is closely intertwined with existing cells and affects the overall IC functionality through a key. If the correct key is given, the IC works as intended. However, for an incorrect key, the IC malfunctions, e.g., producing wrong outputs. A simple example of a locked combinational circuit is shown in Fig. 1. Said key is only known to the design house/ IP-rights holder and is inserted after fabrication into non-volatile on-chip memory. Therefore, in theory, no malicious entity in the supply chain is able to sell overproduced ICs, since they are simply not functional without a correct key. Introduced in 2008 by Roy et al. [10], a dominant strategy is to insert several X(N)OR gates and optionally inverters further down the wires. Simplified, with an incorrect key, the IC will thus “make mistakes” during computations, while the correct key nullifies the modifications.

### Key preprocessors

In the majority of logic locking solutions, all ICs of a design are unlocked with the same key. In turn, as soon as said key is uncovered, all other instances can be unlocked immediately. To mitigate this single-point-of-failure, a *key preprocessor* can be used. This optional module precedes the locking circuitry and derives the key to unlock from a different key that is given to the IC. Through IC-unique values, e.g., derived via a PUF, the input to the key preprocessor is individual for each IC while its output is the same for all ICs. This way, even if the key of the locking scheme is leaked, it cannot be directly used to unlock other ICs.

### Notation and terminology

**Fig. 2:**
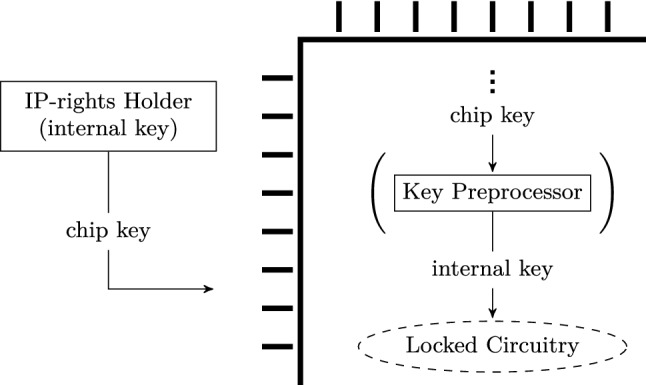
Visualization of the relation between the different keys and elements in logic locking. Note that the key preprocessor is optional

The general terminology in the logic locking literature has not been consistent. Examples for the key that is connected to the locking circuitry include “secret key,” “unlock key,” “master key,” “internal key,” and “chip key.” Below, we compiled a selection of generic terms which appear to be most suitable to address all existing schemes while avoiding confusion. The terms and their relation are also visualized in Fig. 2.

- The *Internal Key* is used by the locking circuitry and only known to the IP-rights holder. While most schemes are unlocked using the same internal key for all ICs, one of the existing schemes inherently unlocks with a different *individual* key for each IC.
- The *Chip Key* is the (external) input to the IC during unlocking. Without a key preprocessor, the chip key is simply the internal key itself. Otherwise, the chip key is the input of the key preprocessor, which in turn computes the internal key.
- *Individual* chip/internal key indicates that the respective key is different for each IC.
- *Global* chip/internal key indicates that the respective key is identical for each IC.

Likewise, the terms “logic locking,” “logic encryption,” and “logic obfuscation” are used synonymously. We want to emphasize a remark from Plaza and Markov that this mixed terminology is ill-advised and in fact misleading [11]. Indeed, “encryption” is tied to making data indecipherable through transformation of the data itself, “obfuscation” transforms a structure into an alternative but functionally equivalent representation, while only “locking” describes key-based restriction of access to functionality. Hence, “logic locking” is notably the most appropriate term.

In addition, presenting logic locking as an obfuscation technique is only partially correct. By definition, obfuscation is a transformation that obstructs comprehensibility but does not alter functionality. Since logic locking introduces an additional input—the key—to the design and changes its functionality based on said input, it is not a plain obfuscation scheme, although it definitely obstructs comprehensibility for a reverse engineer.

## The current state of logic locking

In this section, we give a high-level survey of existing logic locking solutions, followed by a brief overview on existing attacks. This survey forms the baseline of our discussion in Sect. 4.

Recalling Sect. 2.3, a logic locking solution is composed of the locking scheme itself and (optionally) a key preprocessor. Furthermore, two types of locking schemes can be distinguished (cf. Sect. 2.4): almost all schemes incorporate a *global internal key*, i.e., all ICs of that design can be unlocked by the same key. However, one scheme features *individual internal keys*.

For a more in-depth recap of the internals of existing schemes, we refer to the numerous surveys on logic locking, including [12–17].

### Locking schemes with a global internal key

Most locking schemes facilitate a global internal key. The first logic locking scheme was “EPIC” [6, 10]. Here, the locking circuitry, in this case X(N)OR and inverter gates, is randomly inserted into the existing combinational logic. Incorrect key bits result in bit flips in signals, thus leading to faulty computations.

EPIC triggered considerable follow-up work which introduced new schemes with improved placement of the locking circuitry. Rajendran et al. discovered that if inserted at random, parts of the locking circuitry might influence each other and potentially “mute” each other’s effect [18], thus weakening the security of the design. As a result, they presented an algorithm that only inputs a small portion of gates at random while finding optimal positions for the remaining gates. A similar idea is to use fault simulation techniques in order to model the effect a faulty key has on the overall functionality [19]: depending of the position of the locking circuitry, a fault might not be propagated to the outputs. Hence, identifying positions with a good “fault impact” is beneficial for the design to be locked. This strategy has often been used to optimize different locking schemes [20, 21]. In [22], the authors present Strong Logic Locking (SLL). Here, the locking circuitry is inserted such that key bits cannot be deduced from IC output, thwarting a possible reduction of the complexity of brute-force attacks. Combining the ideas presented above, Karmakar et al. introduced an algorithm to determine the optimal position for the locking circuitry [23]. Their logic locking approach is based on fault analysis, while at the same time inserting a part of the key gates according to the rules of SLL.

In addition to finding optimal positions for the locking circuitry, numerous logic locking schemes also focus on the type of locking circuitry. Baumgarten et al. proposed to insert “reconfigurable logic barriers,” i.e., programmable look-up tables, into the design which will be configured after fabrication [7]. The idea to use look-up tables was further enhanced by Yasin et al. in Stripped-Functionality Logic Locking (SFLL) [4, 24]: here, parts of the design functionality are stripped, i.e., missing in the design. A restore unit, consisting of look-up tables in tamper-proof memory, rectifies the faulty behavior if the correct key is present, hence restoring intended functionality. SFLL itself was subject to several follow-up works. One challenge of SFLL is to select which parts to protect: Liu et al. [25] identified a problematic inherent trade-off between security and effectiveness. While a high complexity of SAT attacks is beneficial for a locking scheme, a wrong key should at the same time result in as much error as possible. To optimally balance this trade-off, the authors present Strong Anti-SAT (SAS) as an improvement to SFLL. Another derivative of SFLL presented in [26] extends the original design with one-way functions to thwart the bit-coloring attack introduced in the same work. With ENTANGLE [27], the authors present another variant of SFLL thwarting known attacks by splitting the stripped signals into two groups that are processed separately. Other types of locking circuitry include MUXes [11, 28] to hinder the deduction of key bits, e.g., through test responses (during the testing phase) or by resynthesizing the netlist. [29] makes use of AND resp. OR gates in dedicated locations to also protect against insertion of hardware Trojans.

We emphasize that this broad overview of prior work is not exhaustive, but includes prominent examples of the different variants of logic locking schemes that employ global internal keys.

### Locking schemes with individual internal keys

In contrast to the vast amount of schemes featuring global internal keys (cf. above), there is only a single scheme which generates an individual *internal* key for each IC: CLIP by Griffin et al. [9]. To this end, CLIP employs process variation (PV) sensors which measure slight differences in transistor threshold voltages, yielding a mechanism comparable to a weak PUF. Since it is the only scheme with such a mechanism, we shortly recap it in the following. In order to protect a combinational function *f*, CLIP locally duplicates it. Now, instead of receiving the original inputs, *f* is fed by the output of a so-called switchbox. Input to this switchbox is the original inputs as well as a signal generated by the PV sensor. The switchbox manipulates the original inputs depending on the input of the PV sensor. One possible example of a single-bit switchbox would look as follows: the original input is input to two separate AND functions, the second input to the respective AND function is given by either the original PV sensor’s reading or its inverse. As a result, the switchbox offers two possible input values to the duplicated function *f*, i.e., although equal, both *f*-instances will result in a different output signal. An “output selector,” i.e., a multiplexer, decides, based on one bit of the internal key, which output of the two *f*-instances becomes the final output. As a result, only if the key bit correctly selects the output of the *f*-instance with unmodified inputs, the circuit will behave correctly. With CLIP, each IC produces its own individual internal key, however, even the designer has no way of knowing a specific internal key beforehand. Therefore, the designer has to query each produced chip with a test set in order to recover the internal key from the circuit’s outputs and subsequently unlock the device. The authors acknowledge that this can also be done by a malicious entity.

### Key preprocessors

Since for most locking schemes all ICs share a global internal key, disclosure of said key would immediately enable overproduction and logical IP-theft. The need of a mechanism to individualize key material without adjusting the mask for each IC has been noted by various authors. A key preprocessor can be used to achieve this very goal, i.e., every IC will have an individual chip key from which the internal key is derived on-chip. In general, this derivation is based on an IC-unique value, for example obtained via a physically unclonable function (PUF). This way, despite a scheme having a global internal key, every IC requires its own individual chip key that is according to its PUF response. If the correct chip key of one IC was used to unlock a different IC, the key preprocessor would thus compute an incorrect internal key and the design remains locked. As a direct consequence, even if an adversary obtained the internal key, other ICs would still not be unlockable. While several publications state the necessity of a key preprocessor, only two instantiations have been proposed so far:

Roy et al. presented the first key preprocessor together with EPIC [6, 10].

Note that in the initial version [10], an error in the protocol allowed for an attacker to successfully retrieve the locking key. Maes et al. presented the corresponding attack in 2009 [30]. In the 2010 journal version of EPIC [6], the protocol was fixed in order to thwart this problem. In the following, we refer to this version when describing EPIC’s key preprocessor.

The initial motivation was to enable remote unlocking of ICs via asymmetric cryptography, i.e., the IC does not have to be physically sent back to the design house for unlocking. The key preprocessor features a PUF or TRNG and an RSA engine, capable of key generation, decryption, and signature verification. While the public RSA key of the design house is hardcoded, an individual RSA key pair for the IC is generated during the initial power-up using the PUF or TRNG as a source of randomness. This key pair is then burnt into fuses. In order to unlock an IC, the design house encrypts the global internal key using the individual RSA public key of that IC and then signs the resulting ciphertext with its own private key. The chip key sent to the IC contains both, the encrypted internal key and the signature. The key preprocessor derives the internal key from the chip key by verifying the signature and subsequently decrypting the ciphertext with its own private key. Remote unlocking is enabled since an attacker cannot forge a valid signature.

The idea for a different key preprocessor, a Logic Encryption Cell (LEC), was briefly mentioned by Rajendran et al. in 2012 [19]. For LECs, the chip key consists of a PUF challenge and additional data the authors refer to as *user key*. On chip, the PUF response is XORed with the user key to generate the internal key. With respect to EPIC’s key preprocessor, the authors argue that a PUF circuit can be implemented much smaller than an RSA engine, however, they do not provide any information regarding the concrete instantiation or PUF setup. Furthermore, it is claimed that, even if an adversary obtained the chip key, i.e., PUF challenge and user key, security was not compromised since the attacker cannot guess the PUF response. According to the authors, LECs therefore provide the same security as an RSA engine. However, it is not described how the design house can learn/challenge the PUF prior to unlocking in order to generate valid chip keys.

### Attacks on locking schemes

Logic locking has been subject to several kinds of attacks. In this section, we will briefly introduce the ongoing work in this area, although in less detail than our survey of existing schemes. For more details, we refer the interested reader to [31] and [16].

Most attacks can be characterized as either (1) key extraction attacks, where the adversary tries to uncover the internal key, (2) removal attacks, with the goal of removing the locking circuitry from a locked netlist, or (3) bypass attacks, where the adversary tries to insert bypassing logic into the design that nullifies the locking measure.

The overwhelming majority of attacks on logic locking schemes are based on SAT-solving. The general premise is that an attacker is in possession of the locked netlist, e.g., through theft or reverse engineering, and has obtained an already unlocked device, e.g., from the open market or an insider. Then, the main idea is to find a key which produces the same I/O behavior in netlist simulation as observed from an unlocked device. Alternatively, a locked IC can be used if netlist simulation is infeasible. However, the attacker has to have control over the internal key. Note that the recovered key is not necessarily equal to the actual internal key. The only guarantee is that both keys produce the same outputs for all possible inputs.

The first SAT-based attack on logic locking was presented by Subramanyan et al. [32]. The authors focus on identifying so-called distinguishing input patterns (DIPs), i.e., input/output-patterns that aim to exclude multiple wrong key candidates at once. With respect to locking schemes that insert key gates at random, Subramanyan et al. effectively broke all schemes available at the time.

This result sparked further research in the same direction: with SARLock [33] and Anti-SAT [34], defense mechanisms to thwart SAT-based attacks have been presented shortly after. Prominent examples for attacks against said SAT defenses are the signal probability skew (SPS) attack [35], the novel bypass attack [36], or most recently CAS-Lock [37], which in turn inspired more powerful SAT-based attacks, including Double DIP [38] or the SMT attack [39]. The aforementioned attacks were again followed by even stronger defense algorithms as presented in [40].

Recently, logic locking schemes such as Bilateral Logic Encryption (BLE) [41] have been proposed, stating to rescue locking in the face of current Boolean satisfiability (SAT)-based attacks and their variants. As a result, a new class of SAT-based attacks, Fa-SAT, featuring fault injection techniques into their SAT framework, was developed [42]. Fa-SAT was in turn followed by Distributed Logic Encryption (DLE) [43], a logic locking scheme designed to resist all known attacks *including* Fa-SAT.

Considering the above-mentioned examples, the following becomes obvious: From a high-level perspective, this line of research developed into an arms race between novel attacks against existing SAT defenses and corresponding countermeasures. Crucially, this strong focus led to new weaknesses against other attack vectors: recently, Sengupta et al. demonstrated a critical design flaw in CAS-Lock [5] that allows for trivial unlocking via the all-zero or all-one key.

While the majority of attacks focus on SAT-solving, other attack vectors have received attention as well.

Yasin et al. [8] and Sengupta et al. [44] investigated the effectiveness of side-channel attacks against logic locking. In the first work, Yasin et al. provide experiments which discover more than half of a 32-bit secret key using Differential Power Analysis (DPA) for schemes that insert their key gates randomly. They find that more sophisticated placement of key gates rendered their DPA attacks less efficient. Sengupta et al. extend the aforementioned work by performing further experiments and provide ideas how to thwart side-channel attacks on logic locking in the future. Li et al. introduced an attack that can extract more than half of the bits of the internal key by identifying redundant logic [45] in the locked netlist. Notably, no unlocked IC is needed for this attack. Similarly, experiments show that the so-called functional analysis attack on logic locking (FALL) [46] can defeat logic locking without oracle access in approx. 90% of the cases. The *desynthesis attack* presented by El Massad et al. enables an attacker to discover several bits of the secret key by resynthesizing the design according to the current key candidate [28]. Again, the attacker does not need access to an unlocked IC but precise information on the originally employed synthesis tools and options. In [47], Yang et al. demonstrate how to unlock the supposedly provable secure locking scheme Stripped-Functionality Logic Locking (SFLL) [4] within minutes. Another recent example is the SURF attack by Chakraborty et al. [48], which combines the machine-learning-based SAIL attack [49] with functional analysis in order to successfully recover the internal key.

## Discussing the state of the art

With the survey on available locking approaches, their characterization, and the overview of existing attacks in the previous section, we now discuss similarities but also inconsistencies and shortcomings in the current state of logic locking research.

### Attacker models

The logic locking literature lacks a consistent attacker model. One major problem is that real-world requirements are heavily dependent on application and industry specifics. Unfortunately, thus far, the scientific community lacks insight into companies’ secrets and demands on how to thwart piracy and overproduction. Still, considering logic locking from a scientific point of view, it is equitable not to disregard the capabilities of a potentially strong adversary such as a malicious foundry.

Most publications use the following description of an attacker’s capabilities: the adversary gets oracle access to several locked and unlocked ICs, as well as a gate-level netlist of the locked design. He is allowed to observe the input/output behavior of the ICs *only* via black-box access. Furthermore, he is allowed to analyze and simulate the locked netlist. Note that access to unlocked ICs implies that they are already available on the open market or that the attacker has an insider at some stage where ICs are unlocked.

From a high-level point of view, logic locking aims to defend against a wide range of untrusted actors in the fabrication chain. Contradictory, the attacker model is restrictive and only allows *noninvasive attacks*. The adversary is not allowed to interfere with the fabrication process or open fabricated ICs. However, the latter is clearly possible for virtually any actor in the chain and the former is possible at least for a malicious foundry. Invasive attacks have been mentioned only in passing, cf. [6, 10], but either written off as unrealistic or protection is claimed without an in-depth discussion.

Apart from the description of the adversary’s capabilities, a characterization of the actual protection goals is commonly missing, i.e., protection only against overproduction or also against reverse engineering, etc. Likewise, it is often left unclear which attacks are regarded as successful. For instance, overproduction is *not* immediately enabled by key extraction attacks if a key preprocessor is used (cf. Sect. 3.3). This very problem is also visible in existing work. For example, in [32] the presented SAT attacks claim success after recovering the internal key against EPIC-locked ICs. However, by default EPIC employs its key preprocessor [6, 10], hence recovering the internal key alone might suffice for reverse engineering but not for activating overproduces ICs.

On a general note, considering the malicious foundry as the main threat against logic locking, one can argue that especially with respect to large (profitable) projects, the inhibition level to betray the designer’s trust might be too high. It seems unlikely that the major foundries risk reputation and contracts through piracy. While we do not deny that this is a valid argument, it neglects that besides those global players, there is a large body of smaller foundries all around the world, offering their services for lower-volume or prototyping jobs. Furthermore, the actively ongoing research with respect to logic locking for more than a decade indicates that protection against a potentially malicious foundry is in fact welcome. The significance of this area is also reflected by a considerable body of research that considers foundry-level hardware attacks with respect to hardware Trojans [50–52].

### Success-conditions of existing attacks

Existing attacks rarely discuss their win conditions, i.e., under which conditions the attack is regarded as successful, and the resulting outcome in that scenario. For instance, while the first key preprocessor was already presented along with the first logic locking scheme EPIC, existing attacks on logic locking entirely ignore key preprocessors. In fact, while existing attacks might reveal the internal key, the authors of EPIC already explained that this does not suffice to unlock overproduced instances if their key preprocessor is used. Furthermore, key preprocessors can offer advanced features such as remote authentication via digital signatures in case of EPIC. In turn, these features may enable attacks against the underlying locking scheme, giving another reason to analyze the security of key preprocessors as well.

### Internal key handling

For all existing schemes, upon unlocking, the internal key has to be stored on-chip in non-volatile memory. While most publications do not address storage details, [4] and [13] propose to use read- or tamper-proof memory. However, using dedicated secure memory is not beneficial in the context of logic locking: In typical use cases for protected memory, e.g., to store cryptographic keys, data is read from the protected memory only when needed and cleared from internal registers as soon as possible. The exposure of sensitive data is limited to the bare minimum. However, for logic locking, if the internal key is not available at any point, the IC malfunctions (cf. [21]). This leads to the following generic similarity: Every logic locking scheme will have its internal key constantly available in flip flops (FFs) during operation.

### Individualization of keys

While most schemes facilitate a global internal key, CLIP manages to have individual internal keys. Likewise, by using a key preprocessor, schemes with a global internal key can be enhanced to require individual chip keys. These approaches have to make use of an IC-unique random value. In the examples of CLIP and LECs, a PUF is used while EPIC’s key preprocessor also gives the option to use a classical TRNG. Regardless of the way this (crucial) random value is obtained, it is the only source of individualization. This leads to the following generic similarity: All individualization of key material is based on some kind of internal entropy, e.g., derived from a TRNG or a PUF.

## A revised attacker model

Recall that the primary goal of logic locking is to defend against IC overproduction and subsequent trade, e.g., on the black market (cf. Sect. 2.1). Additional goals commonly include IP protection with respect to reverse engineering. Regarding plain overproduction, the malicious foundry can easily be identified as the main adversary, while reverse engineering can potentially be performed by other parties as well, including competitors or even end users. In this section, we present a novel attacker model for logic locking taking a top-down approach: We formulate our attacker model with the strongest adversary—the malicious foundry—in mind and carefully describe adversarial goals that describe successful attacks.

Depending on their threat model, logic locking schemes can aim to protect only against a subset of our attacker model while still acknowledging the existence of a strong attacker. By comparing said subset to the whole model, security of locking schemes can even be approximately quantified in relation to each other.

### Security assets and attack goals

Our model covers logic locking as a countermeasure to both *physical and logical theft*, i.e., overproduction and reverse engineering. Thus, there are three overarching goals for an adversary to be successful, depending on his target:

1. The adversary is able to unlock arbitrary locked ICs without design modification.
2. The adversary is able to interfere with the fabrication process to disable or weaken the locking scheme, thus enabling to unlock ICs that are produced with the adversary-induced modification.
3. The adversary is able to nullify the locking scheme on netlist level, thus obtaining an effectively unlocked netlist.

If the adversary aims for the primary target of physical overproduction, Goal 1 is not necessarily achieved by recovering the internal key (e.g., through a SAT-based attack) since a key preprocessor may prevent the attacker from generating correct chip keys. If the locking scheme can be removed or bypassed (Goal 2 and Goal 3), the adversary can manufacture unprotected ICs at the cost of mask modification or entirely new mask sets. In the best case for the adversary, he can modify the lithographic masks that were used for the genuine order and start to overproduce (Goal 2) at low cost.

Mask repair techniques are widely used to repair defects in fabricated masks [53, 54]. A more detailed description about mask repairs can be found in Sect. 6.2. Hence, using ready available equipment to change the masks is cost efficient. The only drawback is that the original production should be finished before overproduction since the manipulations are performed on the original mask set.

In the worst case, the adversary has to generate entirely new masks (especially for Goal 3), which is considerably more expensive than production with modified existing masks, cp. Sect. 6.2. Consequently, the threat of these attacks depends not only on technical aspects but also on the financial overhead. For example, a removal attack followed by production of modified ICs may only be worthwhile if the black-market revenue is expected to amortize the production costs of new masks.

Then again, if the adversary follows the target of IP-theft on a logical level, achieving Goal 1 or Goal 2 is not necessarily sufficient, since the IP is not guaranteed to be stripped of the obfuscating elements. Goal 3 however, immediately returns the desired results.

In total, an adequate security assessment of a locking scheme needs to take these factors into account. Since the goals’ implications highly depend on the target, i.e., protection against overproduction and/or IP-theft through reverse engineering, we recommend that schemes clearly state their strength against all three goals and name the targets they want to protect against. Likewise, we recommend that attacks specify which goals are reached in order to assess their effectiveness.

### Adversarial capabilities

Restricting the adversary to black-box access (cf. Sect. 4.1) severely underestimates the physical capabilities of most actors in the fabrication chain, especially foundry-level adversaries who, again, are the main threat regarding overproduction. In our model, the adversary has access to several assets:

- *The gate-level netlist* of the locked design, obtained, for example, via reverse engineering or by translating the GDSII/OASIS files.
- *Multiple locked ICs*, which can be obtained during the regular production process. For behavioral analysis, simulation of the locked netlist may already suffice.
- *Multiple unlocked ICs*, which can be obtained, depending on the scheme, after testing, on the consumer market, or directly in the foundry if remote unlocking is used [6, 10]. We note that in some use cases, e.g., military hardware, obtaining unlocked ICs can be hard or close to impossible.
- *Access to the fabrication*, including artifacts, such as the lithographic masks used to manufacture ICs.
- *State-of-the-art IC analysis equipment*, i.e., testing equipment and tools to perform invasive analysis.

The key advantages of a malicious foundry combine complete knowledge about the design on netlist level with physical access within the manufacturing process. Its extensive insights into the layout of all components ease identification of points of interest. Also, modern foundries need to be equipped with failure analysis, repair, and process debugging tools. Such tools can be easily used to perform invasive attacks such as probing and editing. We note that invasive attacks are not limited to foundry-level adversaries. Other adversaries with the corresponding equipment and expertise can perform similar attacks, albeit potentially at higher effort. We want to emphasize that we do not merely introduce a more powerful adversary, but rather argue that the existing models do not capture the full capabilities of adversaries that target overproduction.

Likewise, note that subsets of these capabilities can describe other actors in the fabrication chain that are weaker/less well equipped than a malicious foundry. If a scheme is secure if only a subset of the capabilities can be used, it is easy to draw a line where protection of the scheme ends. For example, a fictive logic locking scheme that only protects against actors that enter after testing can argue to remove *access to the fabrication* from its constrained adversary.

## Implications of the new attacker model

Our new attacker model allows for several attack vectors which have not been considered in previous work. Especially, *invasive attacks* that go beyond black-box access can (and should) now be considered as well. In the following, we will exemplarily discuss two new attack vectors with broad impact that are enabled in the new model. Note that we do not cover all attack vectors available to an invasive adversary, but rather discuss selected comprehensible but devastating examples. In Sect. 7, we will explain how they are already sufficient to target the underlying mechanics of logic locking in a general manner.

### Attack vector: probing registers

The internal key is a core asset that should only be known to the design house. If an attacker gets hold of said key, the strength of the scheme is notably reduced or even entirely nullified. Hence, a strong attack vector involves probing the internal key of an unlocked IC during operation.

While probing of signal values can be difficult for a generic attacker, modern foundry-level adversaries have access to sophisticated testing labs [55, 56], which are needed, e.g., for failure analysis. Hence, the attacker is well acquainted with probing techniques. If a signal of interest is routed close to the top layer, probing needles can extract signal values quite easily after Focused Ion Beam (FIB) preparation. Note that this routing information is especially readily available to the foundry in form of the GDSII/OASIS files. If a frontside approach is not an option, backside probing techniques can be leveraged, such as e-beam or laser voltage probing as used in standard testing routines [57–59], or electro-optical probing and electro-optical frequency modulation [60]. The latter can be further improved by preprocessing the backside with a FIB [61]. We emphasize again that in addition to the technical capabilities, a malicious foundry has unobstructed insights into the design without the need of expensive and error-prone reverse engineering [62].

However, even with specialized equipment, probing signals faces several difficulties, including fast switching signals and noisy sampling methods. This makes probing dynamic data, e.g., cryptographic keys, a complex and demanding task. However, in the special case of logic locking, the adversary only needs to probe static data (cf. Sect. 4.3) that never changes after power-up. Thus, extracting an internal key or chip key is not temporally restricted, i.e., the adversary does not need control over the clock or other information about the timing of the device under attack, heavily easing the probing process.

### Attack vector: minimal mask modification

Producing lithographic masks is a costly step in modern IC manufacturing.

Unfortunately, defects in masks are very common and significantly decrease the overall mask yield. To avert this problem, mask repair becomes an inherent part of producing masks, and its impact on mask costs has already been studied in 1998 [63]. Over the years, due to shrinking feature sizes, mask repair consequently becomes more challenging. However, making *minimal* adjustments to fabricated masks is still feasible via selected mask repair techniques [61, 64].

**Fig. 3:**
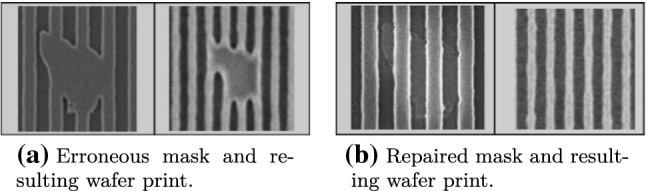
Examples of mask repair [64]

**Fig. 4:**
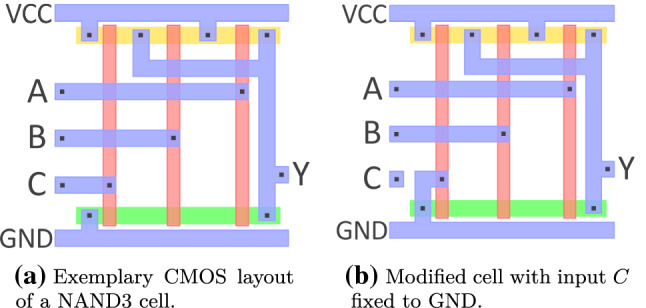
Application of a mask modification attack. Wire spacings and thickness have been adjusted to improve readability. The wire on input *C* was cut and directly connected to GND

In 2006, Lercel and Hector conducted a study on mask repair, concluding that compared to the overall mask fabrication process and its cost, the mask repair step is neither expensive nor time-consuming [65]. Especially compared to the mask writing step, it becomes obvious that repairing a mask is much more cost efficient than producing a new one, as depicted in Figs. 3 and 4 in their study.

Mask repair techniques commonly available to foundries nowadays include e-beams [53, 54, 64] or nano-machining via atomic force microscopy [66], which can even be used beyond 20 nm technology. In 2012, Zeiss, a major provider of such equipment, said that their “current system performance is significantly smaller than the claimed limit of 20 nm.” [66]. Hence, nowadays, it is reasonable to expect that an ever growing number of foundries is capable of performing the aforementioned minimal adjustments. As an example for the effectiveness of minimal mask modification even on small feature sizes, we show in Fig. 3 a mask (32 nm reticle) before and after repair, taken from [64].

We emphasize that, with minimal mask modification, we refer to, for example, cutting selected individual wires at cell inputs and reconnecting them to VCC/GND. We explicitly do **not** require insertion of additional logic cells, which is a significantly more complex task. Minimal modifications can, for example, tie a normally switching signal to a known constant value. The example of connecting a logic-cell’s input wire to VCC or GND is highly feasible, since the respective power rails run directly beneath the cell rows in CMOS. Fig. 4 illustrates this situation by the example of a NAND3 cell: it is evident that only minimal modification is required to connect any of the inputs to GND, as done for input *C* in the example. Note that the same effect could also be achieved with other techniques, e.g., dopant changes in transistors as shown by Becker et al. [67]. In order to stay invisible even under close inspection of the original designer, the malicious foundry could camouflage the gate and instantiate dummy input pins that are indistinguishable from real input pins as demonstrated in [68].

## Invasive attacks on logic locking

In this section, we perform a theoretical evaluation of the attack vectors that are enabled in the revised attacker model against the basic principles of current logic locking schemes. We argue that successful attacks against virtually all logic locking schemes proposed to date are viable. Crucially, an adversary can target the generic properties we outlined in Sects. 4.3 and 4.4. Therefore, the premises of the attacks are applicable regardless of the used scheme. We first discuss how to attack locking schemes without a key preprocessor and subsequently outline attacks on key preprocessors.

### Attacking schemes with global chip keys

We recall that probing of values is readily available to the adversaries considered in our attacker model. This allows an adversary to directly target the internal key. If the locking scheme does not produce an individual internal key for each IC, such a *probing attack* can disclose the global internal key and any overproduced IC can subsequently be unlocked. Note that the vast majority of proposed schemes is solely based on a global internal key (cf. Sect. 3.1).

*Locating the Internal Key* In order to successfully mount such a probing attack, the adversary must be able to locate the internal key signals. Based on our observation that the internal key is constantly available in a register during operation, said register has quite unique characteristics that allow for semi-automated identification. In the following, we will outline approaches on netlist level that help to identify signals for probing.

The first characteristic is that the key is stored in non-volatile memory and loaded into a register through a memory interface during boot. The inputs of said register are only connected to the memory interface, since the register is never loaded with anything else. This largely reduces the search space for the key signals: memory cells are easily distinguishable from logic cells, giving the adversary a starting point for analysis. Tracing the memory wires to subsequent groups of flip flops can be automated in tools like HAL [69]. Furthermore, the register is only loaded during chip initialization. Hence, by analyzing boot behavior, e.g., via dynamic analysis in simulation or by statically evaluating control signals of the netlist, candidate registers can be efficiently filtered. By probing identified registers and then trying the resulting key candidates on a locked IC, the correct key is identified. In addition, the approach of following a memory interface immediately reveals the bit-order of the internal key.

*Attack Characterization* The probing attack aims for Goal 1 and Goal 3 of our attacker model: unlocking arbitrary ICs without authorization and removing the locking scheme on netlist level. While a key preprocessor might thwart reaching Goal 1, Goal 3 is always reached through a probing attack.

Recall that a prerequisite of the attack is that the adversary has access to an unlocked IC. This requirement can be viewed analogously to a known-plaintext attack in cryptanalysis and is consistent with both, our revised attacker model as well as the attacker models used in previous work. As mentioned earlier, unlocked ICs can be obtained, for example, on the open market or from an insider.

Note that locking schemes with individual internal keys are *not* susceptible to mere probing attacks, since obtaining the internal key of one IC does not provide any information on the internal key of other ICs. However, these schemes are inherently vulnerable to minimal mask modification attacks as we will argue in the next section.

### Attacking schemes with individual keys

We recall from our observation in Sect. 4.4 that in order to individualize locking schemes, an entropy source, e.g., a TRNG or a PUF, is required. If it was possible to modify a design such that, instead of the random output, known fixed values were used, the locking scheme would essentially fall back to being deterministic, i.e., using a global key. Note that this does not necessarily disclose sensitive key material but makes all modified ICs unlock with the same chip key.

**Fig. 5:**
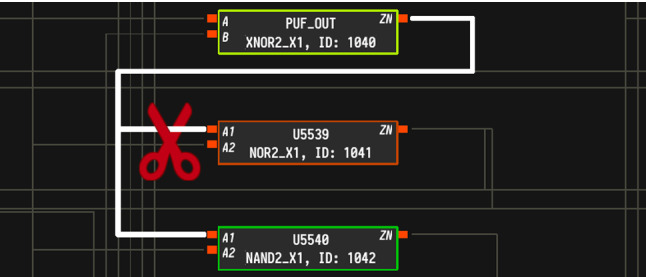
Visualization of a wire split in HAL. The red scissor marks a safe position for wire cutting to affect only the orange cell

*Attack Strategy* Naively modifying an entropy source to output constant values would quickly trigger self-tests and potentially result in unusable ICs. Hence, the modification has to affect only the locking circuitry and avoid other signals in the design. A strategy to achieve this behavior is visualized in Fig. 5. Suppose that the yellow cell outputs the result of a PUF, the orange cell eventually connects to the key register, and the green cell belongs to a different module, e.g., a self-test. If the outgoing wire (highlighted in white) was cut at the position of the red scissor, only the orange cell is affected and the green cell still gets the original PUF output. More precisely, by modifying the input pin A1 of cell U5539 and connecting it to GND or VCC, the key register will receive static input while all other modules that use the PUF, e.g., a self-test, are *not* impacted by the modification. As discussed in Sect. 6.2, forcing a signal to GND or VCC is particularly easy with minimal mask modification by reconnecting the inputs of selected standard cells to the nearby power rails.

*Locating the Signals of Interest* Similar to finding the key register, the adversary needs to identify the location at which the mask modification has to be applied. In general, the entropy source (that is, in practice either a TRNG or a PUF) itself can be identified due to its specific structure, which is largely different from the structures employed in the remaining IC. Examples include analog components within digital logic, combinational loops used for ring-oscillator PUFs or TRNGs, or transistor groups that do not match any standard cells such as the PUF structure from [9].

We now give a strategy how such a position can be determined semi-automatically. The idea is to trace output wires of the entropy source until a key register or a subsequent gate is hit. Then, the respective gate’s input is tied to VCC or GND (attacker chosen). In detail, first, the internal key signals are identified with the same strategy as for the aforementioned probing attack. Then, succeeding gates are traced up to a certain depth. All gates along the traversed paths are marked as *key-affected gates*. Second, the outputs of the entropy sources are traced forward until a key-affected gate is hit. The identified input pin of the key-affected gate is marked for modification as shown in Fig. 4. The given strategy is applicable to circuits where the key is computed/derived from the entropy source and to circuits where the entropy source and the key are both inputs to converging combinational logic cones.

*Attack Characterization* All ICs produced with the modified masks are identical clones of each other with respect to the locking scheme, since any chip-unique random input has been changed to a constant. Still, the ICs are operational since the modification does not affect unrelated modules like self-tests. An adversary can now unlock all subsequently fabricated ICs with a global key. Note that, in general, the adversary does not need to select a specific value for this constant since his goal is just to remove the “uniqueness” of each IC.

Hence, the mask modification attack aims for Goal 2 of our attacker model: disabling the locking scheme during fabrication. In the special case of the CLIP scheme, the attack also sets the internal key to an attacker-chosen value, hence also reaching Goal 3. A mask modification attack is particularly effective if the ICs are ordered in a single batch, as it is often the case in military products. In that case, mask modification can be performed after all genuine chips have been shipped and the legitimate designer will never receive modified instances. As a side effect, in such a scenario, the adversary does not even need to obtain any unlocked ICs.

As mentioned before, reconnecting a pin to VCC or GND, dopant changes, and camouflaging are just a few examples of how to facilitate the attack. Note that the general approach does not rely on locking scheme details, but solely on the fact that random signal values can be turned into constants with mask modification without impacting IC functionality outside of logic locking.

### Attacking key preprocessors

As noted in Sect. 3.3, employing a key preprocessor is advantageous because it can retain security even if the internal key is disclosed. While this is true when considering only noninvasive attacks, in the following we argue that no existing key preprocessor protects against a malicious foundry capable of invasive attacks. We recall that, in previous work, two key preprocessors have been presented, namely the EPIC key preprocessor [6, 10] and LECs [19].

#### LECs

LECs make use of a PUF to facilitate individual chip keys. However, when working out the specifics, several weaknesses arise. The only details given in [19] are that there is a PUF which receives a challenge and outputs a response (cf. Sect. 3.3). This mirrors the structure of a strong PUF, although no helper data is mentioned, which is typically needed in the reconstruction step of a strong PUF. The PUF output is XORed to the so-called user key to generate the internal key. Hence, in order to construct a valid user key, the design house has to know the PUF response for the chosen challenge, i.e., an interface to query the PUF is required. However, an adversary that is already in possession of the internal key could use this very interface in a similar way for an attack that targets unlocking of arbitrary locked ICs (Goal 1). Alternatively, without knowledge of the internal key, the adversary can model the PUF of each IC via machine learning [70, 71]. He can then obtain an unlocked IC and use the corresponding model to compute the PUF response for the respective IC’s challenge. This in turn reveals the internal key through an XOR with the user key and enables the aforementioned attack.

We emphasize that these attacks are based on our assumptions regarding the PUF instantiation. Unfortunately, [19] does not provide definite detail, hence we cannot elaborate further in this case. However, the mere application of a PUF is directly vulnerable to a mask modification attack, comparable to our case study with CLIP (cf. Sect. 7.2).

#### EPIC’s key preprocessor

We recall that the EPIC key preprocessor uses asymmetric cryptography to establish an individual chip key for each IC, even if the underlying locking scheme incorporates a global internal key. Furthermore, the chip key is comprised of the encrypted internal key and a digital signature for authentication.

We will now outline multiple exemplary attacks, based on the generic attack vectors probing and mask modification. We first analyze the potential of each attack vector individually, before evaluating them in a combined fashion. Note that while these are the first attacks that even consider EPIC’s key preprocessor, most other logic locking scheme fail to develop their own preprocessor and merely refer to EPIC. Hence, it is worth mentioning that making use of a key preprocessor in the first place is a strength of EPIC, not a weakness.

*Probing Attacks* EPIC provides a high-level information on how the unlocking procedure works, but it is not clear whether the chip key is stored directly on-chip and the key preprocessor is invoked with each power-up, or whether only the derived internal key is stored after initial unlocking. However, in both cases, a probing attack against EPIC’s key preprocessor can eventually reveal the internal key which reaches Goal 3 of our attacker model. If only the internal key is stored, it can be probed directly. If the chip key is stored, the internal key can in turn be probed from the key preprocessor’s output wires. Similar to the probing attacks in the previous section, the main obstacle for an adversary is to identify the signals of interest. Another viable approach is to extract the chip key itself as well as the IC’s internal RSA key pair, by probing the fuses (as shown for eFuses on FPGAs in [72]). This key pair can then be used to obtain the internal key by decrypting the chip key.

However, knowledge of the internal key alone is not sufficient to directly unlock other ICs (Goal 1) due to the digital signature as already outlined by the authors of EPIC [6, 10]. Crucially, we will show that a combination of probing attack and mask modification attack is indeed successful to achieve physical overproduction.

*Mask Modification Attacks* A mask modification attack on the EPIC key preprocessor can target the randomness that is used to generate the internal RSA key. This will lead to a situation where all ICs use the same (attacker chosen) key pair and ultimately accept the same chip key. Alternatively, the same behavior can also be achieved by targeting the fuses where the RSA key pair is stored: by fixing the output signals of said fuses to constant values on mask level, all ICs again share the same key pair. Without valid chip keys, this attack alone does not fully break the scheme, though. However, recall that a motivation for EPIC’s key preprocessor was remote unlocking, where upon request the design house transmits a valid chip key for an ICs. By requesting to unlock a single modified IC the attacker can then unlock all “clones” of that IC with the same data, no probing required, reaching Goal 2 of the attacker model.

A different target for an attack is the signature verification mechanism used to authenticate the chip key. No matter how the verification is implemented, it eventually comes down to a binary decision of accept/reject. By forcing this signal to always-true, every chip key passes signature verification. Note that this only disables authentication, not the locking mechanism itself and that this attack alone does not fully break the scheme.

*Combining Attacks* As shown above, mask modification attacks can successfully disable the benefits of EPIC’s key preprocessor if remote unlocking is used. If unlocking is performed solely back at the design house, none of the aforementioned attacks alone suffice for a successful attack. However, combining mask modification with an attack that discloses the internal key invalidates the EPIC key preprocessor even in that scenario. Once the key has been obtained, e.g., through probing attacks, the adversary has two options: he either fixes the input signals to the internal key register to the extracted internal key, or, with only a single modification, disables signature verification which enables the forgery of chip keys knowing the internal key. Both options reach Goal 2 and Goal 3 of the new attacker model. The attack vectors for both scenarios are summarized in Table 1. While in [6, 10] an attack that modifies masks was regarded as unrealistic, we argued in Sect. 6.2 that minimal mask modification is not only a viable technique but also a widely used method in modern semiconductor manufacturing and especially applicable to manipulate standard cell inputs/outputs.

**Table 1:** Summary of required techniques to facilitate attacks against EPIC’s key preprocessor

Attack	Unlocking scenario	Reached goals
Probing	Remote	Goal 3
	In-house	Goal 3
Mask modification	Remote	Goal 2
	In-house	–
Combined	Remote	Goals 2 and 3
	In-house	Goals 2 and 3

## Discussion

Our main finding is that the security of logic locking is at risk when invasive attacks are taken into account. Crucially, because of the way current logic locking schemes are designed, probing and mask modification attacks are *always* applicable to logic locking.

**Table 2:**
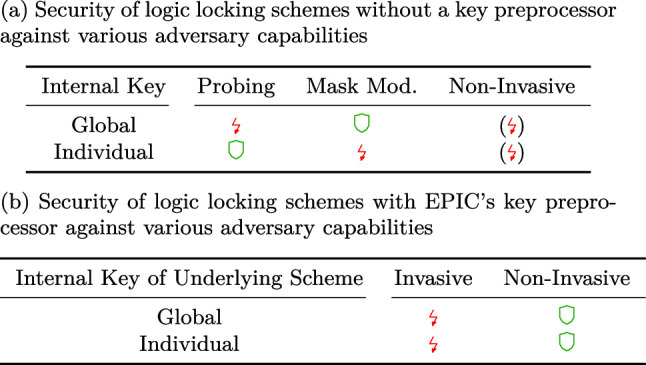
Summary of the security of logic locking with respect to various adversary capabilities

Legend:  = *not vulnerable*,  = generically *vulnerable*, () = ongoing race between attacks and countermeasures

We argued that, in absence of a key preprocessor, all existing locking schemes can be successfully attacked with at least one of the discussed attacks. While the details of attacks may somewhat vary depending on the cell library or design architecture, the underlying idea is applicable to any locking scheme. Even if new logic locking schemes that are perfectly secure against all noninvasive attacks (such as SAT-based attacks) were discovered, a global internal key is always vulnerable to a probing attack. The same holds for mask modification attacks with respect to the discussed attacks. This result is summarized in Table 2. Hence, there are strong indications that, without a key preprocessor, none of the available schemes are secure against overproduction.

Note that specific countermeasures against the discussed attacks may be crafted, e.g., using active shields or a backside polishing detector to thwart probing, as proposed by Rahman et al. [73]. However, most of these countermeasures are merely a specific raise-the-bar measure, and the fundamental security problems remain.

When taking key preprocessors into account, the situation slightly changes. In contrast to plain locking schemes, noninvasive attacks have not been applied to successfully attack a key preprocessor. However, we showed that existing key preprocessors can be attacked in multiple ways when invasive techniques are taken into account by targeting our basic observations from Sect. 4. As a result, Table 2 highlights our conclusion that regardless of the underlying locking scheme, invasive attacks are likely able to fully circumvent EPIC’s key preprocessor.

*Generalizing Our Findings* Our discussions demonstrate that for current schemes, IP-theft on logical level, i.e., reaching Goal 3 of the attacker model, is always possible with a probing attack, regardless of a potential key preprocessor. This also raises the question whether it is possible to thwart overproduction with the current logic locking approaches at all. We acknowledge that protection schemes usually aim to raise the bar for attacks, i.e., render the attack impossible for most attackers. This is most certainly true for a single rogue employee. Still, we showed that current logic locking schemes fail to counter overproduction in the presence of a determined malicious foundry. Hence, logic locking is not as powerful as typically claimed.

To this end, we argued that schemes which use only global internal keys are always vulnerable to probing attacks. Likewise, randomness that is used to individualize key material can always be meaningfully modified via mask modification in a way that only the locking circuitry is affected. This can even be done stealthily with gate camouflaging, invisible to the designer.

From an adversary’s point of view, making use of mask repair techniques in order to perform minimal mask modification attacks allows for an extremely cost-efficient attack. The adversary can use the existing masks with only the small monetary overhead of “repairing” them instead of manufacturing a whole new expensive set of masks. Hence, even when selling a small number of ICs on the black market, the adversary can already make a profit. Moreover, in theory, the adversary would even be able to produce an unlimited amount of black-market ICs from the one set of masks, i.e., depending on the demand, the attack could quickly become quite profitable.

Notably, a malicious foundry has full access to an error-free netlist, and knows many other details about the target IC. We argued that regions of interest are either key registers or entropy sources, both of which exhibit unique characteristics which further ease identification. Hence, foundry-level invasive attacks appear to be a major threat against the underlying aspects of both, locking schemes and key preprocessors, in all available configurations.

## Conclusions

The starting point of this work was a comprehensive summary of the current state of logic locking research. We identified underlying similarities of existing schemes and highlighted shortcomings with respect to attacker models, notation, and attack focus. Looking at the range of adversaries that logic locking aims to defend against, we found that the attacker model(s) of previous work are insufficient to capture the actual physical capabilities of adversaries. Especially a malicious foundry, as the main threat regarding overproduction, is capable of a variety of invasive attacks in addition to the noninvasive attacks considered in previous work.

We introduced a novel attacker model that also captures the physical capabilities of malicious entities in the fabrication chain. Our model includes definitions for several kinds of successful attacks, enabling a more precise assessment and even a relative quantification of locking schemes. Based on the new model, we exemplarily explored two invasive attack vectors, probing and minimal mask modification. Backed by academic works of the specific areas, we argued that both techniques are in fact applicable. We outlined exemplary invasive attacks that enable a malicious foundry to attack virtually all available locking schemes and even invalidate the benefits of the current state-of-the-art key preprocessor from [6, 10].

**Table 3:**
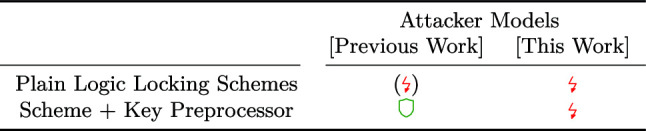
Condensed overview on the security of logic locking against invasive and noninvasive attacks

Legend:  = *not vulnerable*,  = generically *vulnerable*, () = ongoing race between attacks and countermeasures.

Table 3 aggregates our results. Crucially, no combination of scheme and key preprocessor seems to provide sufficient security against a malicious foundry.

From our perspective, logic locking is successful in raising the bar for most attackers although not as high as oftentimes claimed. ICs that are not available on the open market, e.g., designed for special secret military purposes, can be somewhat secured from reverse engineering and overproduction (if the original foundry can be trusted): even if the GDSII/OASIS files are intercepted by a malicious third party, without access to any (un)locked ICs performing any invasive attacks is not feasible. Hence, if we find ourselves in a scenario where the adversarial capabilities are limited to black-box access, the main proposition of logic locking holds. However, and this is the crucial take-home message, given the results of this work, it seems that logic locking is an insufficient countermeasure in the presence of invasive attacks. While raising the bar for successful attacks, a determined malicious foundry is fully equipped to perform the necessary steps in order to facilitate piracy of ICs.

Hence, we argue that research on logic locking needs a fundamental rethinking in its core mechanisms to stay strong, even when faced with such powerful adversaries.

## Data Availability

Data sharing is not applicable to this article as no datasets were generated or analyzed during the current study.
